# The impact of anxiety and affective disorders on breast cancer screening in women in Puerto Rico

**DOI:** 10.1371/journal.pone.0357375

**Published:** 2026-09-11

**Authors:** Marjorie Vázquez-Roldán, Karen J. Ortiz-Ortiz, Liza I. Millán-Pérez, Axel Gierbolini-Bermúdez, Ruth Ríos-Motta

**Affiliations:** 1 Division of Cancer Control and Population Sciences, University of Puerto Rico Comprehensive Cancer Center, San Juan, Puerto Rico; 2 Department of Health Services Administration, Graduate School of Public Health, Medical Sciences Campus, University of Puerto Rico, San Juan, Puerto Rico; 3 Auxiliary Secretariat for Public Health Surveillance & Protection, Puerto Rico Department of Health, San Juan, Puerto Rico; Local Health Authority Caserta: Azienda Sanitaria Locale Caserta, ITALY

## Abstract

**Background:**

Breast cancer is the most common cancer among women globally. Early detection using mammography is crucial for improving outcomes and reducing aggressive treatment. Factors such as age, comorbidities, insurance coverage, and mental health conditions may influence screening behavior. Limited research exists on the relationship between mental health and breast cancer screening in Puerto Rico. This study examines factors associated with early detection among women aged 50 and older diagnosed with breast cancer and coexisting anxiety or affective disorder.

**Methods:**

We conducted a retrospective cohort study using data from the Puerto Rico Central Cancer Registry linked with the Health Insurance Linkage Database. Multivariable logistic regression was used to estimate adjusted odds ratios (AOR) to describe the relationship between having anxiety or affective disorder with the use of mammography and the stage at diagnosis. Cox proportional hazards model was used to assess the association between mental health diagnosis and risk of death, respectively.

**Results:**

Among 4,708 eligible women, 376 (8.0%) had an anxiety or affective disorder. Overall, 90.0% received a mammogram within 24 months prior to diagnosis, and 35.1% were diagnosed at a late stage. Women with anxiety or affective disorder were 57.0% more likely to have undergone screening (AOR 1.57, 95% confidence interval [CI]: 1.01–2.43; p = 0.046). These disorders were not significantly associated with cancer stage at diagnosis (AOR 0.86, 95% CI 0.68–1.09; p = 0.208) and did not increase the risk of death within 5 years (Adjusted Hazard Ratio [AHR] 1.01, 95% CI: 0.76–1.35; p = 0.094).

**Conclusion:**

In our study, anxiety or affective disorders were associated with increased mammography use but not with earlier cancer stages. Further research is needed on severe mental illness and the bidirectional relationship between cancer and mental health.

## Introduction

Breast cancer is the leading type of cancer impacting women worldwide [1,2]. Delay in diagnosis hinders women’s possibilities of less aggressive cancer treatment and survival. Women living with mental health conditions have been found to have worse health outcomes, including less adherence to screening guidelines, involvement in risky practices, and higher mortality [3]. A delay in cancer diagnosis may impact morbidity and mortality, as it represents the possibility of identifying the condition at a more advanced stage, requiring a more aggressive treatment plan [4].

Timely screening is one of the most effective measures for reducing both morbidity and mortality associated with breast cancer [5–7]. Mammography is the screening tool that has been associated the most with breast cancer survival [8,9]. While there is a clear understanding of the benefits of using mammograms for breast cancer screening, there are factors that may constitute barriers to access. These factors include age [10–12], having no health insurance, or having Medicaid coverage [13,14], lack of social support [10,15], limited geographic access to screening services facilities [16–18], as well as clinical factors to consider, such as biomarkers and comorbidities [19]. Concerning health insurance, despite Puerto Rico’s economic crisis and healthcare challenges, only 6% of the population lacks health coverage [20]. Moreover, medically indigent patients at high risk of chronic conditions, such as cancer, have access to health coverage through Medicaid [21]. However, it has been shown that cancer patients, including women with breast cancer, who are Medicaid enrollees, experience worse health outcomes [13,14,22]. These factors constitute challenges in breast cancer detection and outcomes.

Comorbidities, including mental disorders, are barriers that impact access to screening, but there is no certainty of the reach of their influence [23]. In some cases, comorbidities have been shown to promote a surveillance effect, helping to identify and treat illnesses in a timely manner [23,24], given that women are in the healthcare system, which increases the chances of accessing screening according to guidelines. On the other hand, comorbidities may create a distracting effect, deterring correct identification of signs and symptoms and leading to what has been identified as missed opportunities in primary care [25] and to late diagnoses [23].

Mental health conditions, such as depression and anxiety, can develop at any time during the cancer care continuum [1]. These may act as barriers to timely care and cancer screening [26], adversely affecting the chances of early diagnosis and decrease mortality of women with breast cancer [26–28]. Even if the incidence of cancer between women diagnosed with mental health conditions and the general population is similar, they experience increased mortality and poor quality of life [29]. It is estimated that during their lifetime, 1 in 3 women will experience depressive disorders [30]. Anxiety disorders are characterized by a sentiment of nervousness, irritability or hostility, among others, about everyday life matters [30]. It is estimated that between 2 and 11% of women suffer anxiety at any time during the past year, worldwide.

In Puerto Rico, according to the Department of Health, from July 2022 to June 2023, a total of 17,011 patients received services from mental health programs or substance abuse programs; of those, 12,750 were men and 4,065 women [31]. Canino et al (2019) estimated that the prevalence of mood disorders in Puerto Rico was 9.9% and 12.5% for anxiety disorders [32]. In 2021, the estimated prevalence of depression in Puerto Rico for women was 14.7% [33]. However, there is limited statistical information about anxiety or other disorders and other comorbidities to mental health besides substance abuse. Moreover, there is a significant gap in knowledge regarding the impact of mental health disorders in Puerto Rico. Particularly, on how these conditions interact with other health outcomes, such as cancer screening and overall healthcare access [34]. Studies about breast cancer screening in Hispanic women in Puerto Rico are more limited [35]. This study aims to explore the prevalence of anxiety disorders or affective disorders among women diagnosed with breast cancer. In addition, we evaluated whether there is a relationship between having a diagnosis of anxiety disorders or affective disorders and the use of breast cancer screening tests, mammography to be specific, in women aged 50 and over. Considering that adherence to screening is affected by other factors, the study also assessed factors that influenced access to mammography in women with a diagnosis of anxiety or an affective disorder and their impact on survival.

## Methodology

### Design and data source

This retrospective cohort study was conducted using de-identified data from the Puerto Rico Central Cancer Registry, linked with the Health Insurance Linkage Database (PRCCR-HILD) [36]. Fully anonymized data was first accessed on March 20, 2024. The registry is the cancer epidemiological surveillance system in Puerto Rico, which was established in 1951. The PRCCR adheres to the standards established by the National Program of Cancer Registries (NPCR) and the North American Association of Central Cancer Registries (NAACCR). These two entities are responsible for oversight to ensure that the data complies with the highest standards of completeness and precision [36]. Claims are rendered by insurance companies, including Medicaid, Medicare, and Private Health Insurance.

The study was reviewed and approved by the Institutional Review Board (IRB) of Human Research Subjects Protection Office of the University of Puerto Rico, Medical Sciences Campus. The IRB determined that informed consent was not required (IRB Id. 2310157056, Date October 26, 2023).

### Cohort definition

**Fig 1:**
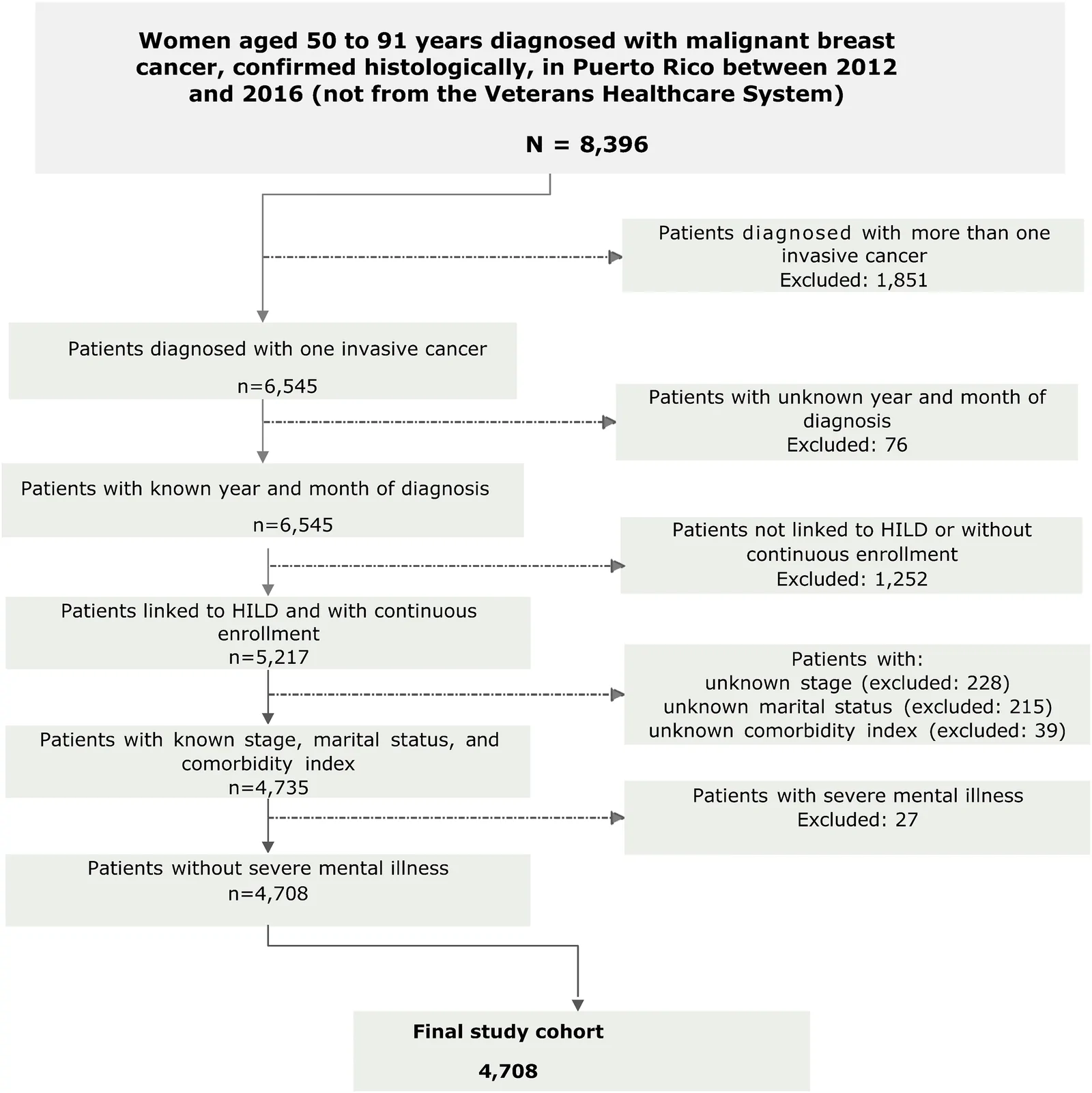
Population Study Selection Flowchart.

Cohort selection is described in Fig 1. We included women aged 50–91 years diagnosed with malignant breast cancer, confirmed histologically, residents in Puerto Rico between 2012 and 2016. Exclusion criteria included women with multiple cancer diagnoses, missing information regarding comorbidities, marital status, or stage at diagnosis. Additionally, cases that did not have continuous enrollment for 24 months before the diagnosis were excluded. Concerning mental health disorders, cases diagnosed using codes F00-F29 and F60-F99 from ICD-10 were excluded from the sample.

### Variables

#### Main predictor.

For anxiety and affective disorders, the study team looked at claims within 12 months prior to cancer diagnosis, using CPTs for psychosocial evaluation or services (among others). Additionally, for diagnostics using the International Statistical Classification of Diseases and Related Health Problems 9^th^ and 10^th^ Revision (ICD-9 and ICD-10) was used. Codes from ICD-10 describing anxiety disorders (F40-48), affective (also described as mood) disorders (F31-F39) were included.

#### Outcome variables.

The use of screening services, such as undergoing a mammogram within 24 months before receiving the cancer diagnosis, was categorized as *Yes* for those who had mammogram claims and *No* for those who did not. Stage at diagnosis was defined using the SEER Staging Manual as localized, regional, or distant/metastatic. For analysis purposes, the stage was defined as early stage (localized) and late stage (regional and distant/metastatic). For risk of death within 5 years, the outcome was defined as time from cancer diagnosis to death from any cause. Patients alive at the end of the study period were censored at the follow-up date (5 years since the diagnosis).

#### Independent variables.

Age at diagnosed was divided into 3 categories: 50–59 years; 60–69 years and ≥70 years. Marital status was categorized as married (legally married or common law arrangements) and unmarried; it was also obtained from records. Insurance coverage was defined as users of: Medicare, dual coverage Medicare-Medicaid, Medicaid, and private health insurance. It is important to mention that cases of women without health insurance were not included in the sample. The health region of residence categories was defined by dividing the 78 municipalities into the 7 regions, Puerto Rico’s Health Department. Finally, biomarkers were divided into three categories: ER/PR+ (Positive Hormone Receptor) and HER2 (ERBB2) Negative for Epidermal Growth Factor (HER2-); Triple Negative (ER-, PR-, HER2-) and other combinations or unknown.

### Statistical analysis

The data analysis included univariate analyses utilizing descriptive statistics such as means, percentages, and frequencies. Covariates were selected a priori based on clinical relevance, prior literature, and availability in the cancer registry data. Simple logistic regression and multivariable were used to estimate crude and adjusted odds ratios (OR) to describe the relationship between the diagnosis of mental health with the use of mammography and the stage at diagnosis. Cox proportional hazards model was used to assess the association between mental health diagnosis and risk of death, respectively. Prior to conducting multivariate analyses, the likelihood ratio tests were applied to evaluate potential interactions between the independent variables. Adjusted Odds Ratios (ORs) and Hazard Ratios (HRs) and their 95% confidence intervals (CI) with the statistical significance were determined at the p = 0.05 level. Statistical analyses were completed using STATA version 17 (Stata Corp, Texas, USA).

## Results

### Clinical and sociodemographic characteristics.

The study population consisted of 4,708 individuals who met the inclusion criteria and were diagnosed with cancer in Puerto Rico between 2012 and 2016. Notably, 4,332 (92.0%) did not have any claims for mental health services in the twelve (12) months prior to their breast cancer diagnosis. Of the 376 women (8.0% of the total) who had a mental health condition diagnosed as identified by claims, 309 were related to a diagnosis of depression, 76 had a diagnosis of anxiety, and 16 of them presented a diagnosis of bipolar disorder, showing some overlap among conditions. Demographic characteristics are shown in Table 1.

**Table 1:** Demographic characteristics of the study population.

Characteristics	N = 4,708	Percentage
**Age**		
50-59 years	1,400	29.74
60-69 years	1,695	36.00
≥70	1,613	34.26
**Marital status**		
Married	2,414	48.73
Unmarried	2,294	51.27
**Health insurance**		
Medicaid	1,180	25.06
Medicare	1,651	35.07
Medicare-Medicaid	757	16.08
Private	1,120	23.79
**Health Region of Residence**		
Metro	1,096	23.28
Arecibo	603	12.81
Bayamon	663	14.08
Caguas	705	14.97
Fajardo	173	3.67
Mayagüez	767	16.29
Ponce	701	14.89
**Comorbidity Index**		
0	3,110	66.06
1	854	18.14
≥2	744	15.80
**Biomarkers**		
ER/PR + Her2-	2,280	48.43
Triple negative	454	9.64
Other combinations or unknown	1,974	41.93
**Anxiety or Affective Disorde**r		
Yes	376	7.99
No	4,332	92.01
**Mammography (24 months)**		
Yes	4,237	90.00
No	471	10.00
**Stage at diagnosis**		
Early	3,058	64.95
Late	1,650	35.05

Of the study population, 4,237 (90.0%) had undergone breast cancer screening, defined by having at least one mammogram in the 24 months before their breast cancer diagnosis. On the contrary, 471 (10.0%) women had not had a mammogram. In terms of the stage at the time of cancer diagnosis, 35.1% (n = 1,650) of the women had a late-stage diagnosis.

### Factors associated with screening.

It was observed that having an affective or anxiety disorder was associated with undergoing a mammogram (p < 0.05) (Table 2). In the adjusted model, women with diagnoses of anxiety and affective disorders were 57.0% more likely to have a mammogram than those without a mental health diagnosis (AOR = 1.57, 95% IC: 1.01, 2.43; p = 0.046). The likelihood of undergoing mammography decreased with age and showed statistical significance for the age group of 70 years or older (AOR = 0.47, 95% CI: 0.35, 0.63; p < 0.001). Enrollment in private or Medicare health insurance increased the likelihood of getting a mammogram, compared to being enrolled in Medicaid. Dual coverage of Medicare and Medicaid, enrollees as well as Medicare, or private insurance enrollees were between 52.0% and 65.0% more likely to have a mammogram as established by the guidelines, compared to Medicaid enrollees. The comorbidity index also showed statistical significance with having undergone mammography (AOR = 1.99, 95% CI: 1.39, 2.66, p < 0.001).

**Table 2:** Factors influencing mammography use in the 24 months prior to breast cancer diagnosis in a sample of women aged 50 years or older in Puerto Rico during the period 2012-2016.

	Unadjusted OR		Adjusted OR	
**Factor**	**[95% CI]**	**P value**	**[95% CI]**	**P value**
**Anxiety and Affective Disorders** (Reference: No)				
Yes	1.77 [1.15, 2.73]	0.01	1.57 [1.01, 2.43]	0.046
**Age** (Reference: 50–59)				
60-69	0.84 [0.66, 1.08]	0.173	0.69 [0.53, 0.90]	0.005
≥ 70	0.71 [0.55, 0.90]	0.005	0.47 [0.35, 0.63]	<0.001
**Marital status** (Reference: Unmarried)				
Married	1.23 [1.01, 1.48]	0.037	1.12 [0.92, 1.37]	0.253
**Health Region of Residence** (Reference: Metro)				
Arecibo	0.95 [0.69, 1.29]	0.732	0.92 [0.67, 1.27]	0.619
Bayamon	1.62 [1.14, 2.29]	0.007	1.64 [1.15, 2.34]	0.007
Caguas	1.17 [0.85, 1.59]	0.394	1.15 [0.83, 1.58]	0.394
Fajardo	0.87 [0.53, 1.41]	0.566	0.92 [0.56, 1.51]	0.749
Mayagüez	1.18 [0.87, 1.61]	0.277	1.17 [0.86, 1.60]	0.310
Ponce	1.26 [0.92, 1.73]	0.157	1.26 [0.91, 1.75]	0.159
**Health Insurance** (Reference: Medicaid)				
Medicare	1.65 [1.30, 2.10]	<0.001	1.89 [1.43, 2.49]	<0.001
Medicare-Medicaid	1.56 [1.16, 2.11]	0.003	1.60 [1.16, 2.22]	0.005
Private	1.62 [1.24, 2.11]	0.003	1.50 [1.15, 1.98]	0.003
**Biomarkers** (Reference: ER/PR + Her2-)				
Triple Negative	0.75 [0.54, 1.04]	0.083	0.75 [0.54, 1.04]	0.079
Other combinations or unknown	0.79 [0.64, 096]	0.020	0.76 [0.61, 0.93]	0.008
**Comorbidity Index** (Reference: 0)				
1	1.94 [1.44, 2.60]	<0.001	1.99 [1.47, 2.68]	<0.001
≥2	1.85 [1.36, 2.52]	<0.001	1.92 [1.39, 2.66]	<0.001

### *Factors associated with late stage*.

Table 3 shows the results of the analyses describing the relationship between factors of access to services and late-stage breast cancer in women over 50 years of age between 2012 and 2016. The principal study variable, having a diagnosis of affective and anxiety disorder, did not show a statistically significant association with an advanced stage at the time of diagnosis (p < 0.05). Married women were 15.0% (AOR = 0.86, 95% CI: 0.76, 0.97; p = 0.015) less likely than unmarried women to be diagnosed at a late stage. As well as both Medicare, dual Medicare/Medicaid, enrollees were more likely to be diagnosed at an early stage (p < 0.05). Having a mammogram in the 24 months before the diagnosis of breast cancer decreases the likelihood of having a late diagnosis by nearly 50% (AOR = 0.49, 95% CI: 0.04–0.60; p < 0.001).

**Table 3:** Factors influencing late-stage breast cancer diagnosis in a sample of women aged 50 years or older in Puerto Rico during the 2012-2016.

	Unadjusted OR		Adjusted OR	
**Factor**	**[CI 95%]**	**P value**	**[CI 95%]**	**P Value**
**Anxiety and Affective Disorders** (Reference: No)				
Yes	0.85 [0.68, 1.06]	0.15	0.86 [0.68, 10.9]	0.208
**Age** (Reference: 50–59)				
60-69	0.72 [0.63,0.84]	<0.001	0.81 [0.69,0.95]	0.009
≥70	0.76 [0.65, 0.88]	<0.001	0.90 [0.75, 1.09]	0.269
**Marital Status** (Reference: Unmarried)				
Married	0.85 [0.75, 0.95]	0.006	0.86 [0.76, 0.97]	0.015
**Health Region of Residence** (Reference: Metro)				
Arecibo	0.90 [0.73, 1.11]	0.331	0.90 [0.73, 1.12]	0.336
Bayamon	0.95 [0.77, 1.16]	0.593	0.92 [0.75, 1.13]	0.420
Caguas	0.88 [0.72, 1.06]	0.201	0.91 [0.74, 1.11]	0.357
Fajardo	0.96 [0.69, 1.34]	0.795	0.92 [0.66, 1.30]	0.648
Mayagüez	0.78 [0.64, 0.95]	0.013	0.77 [0.63, 0.94]	0.009
Ponce	1.02 [0.84, 1.24]	0.874	0.99 [0.81, 1.22]	0.946
**Health Insurance** (Reference: Medicaid)				
Medicare	0.59 [0.50, 0.69]	<0.001	0.65 [0.54, 0.78]	<0.001
Medicare-Medicaid	0.65 [0.54, 0.79]	<0.001	0.73 [0.59, 0.90]	0.003
Private	0.82 [0.69, 0.97]	0.021	0.86 [0.72, 1.02]	0.09
**Biomarkers** (Reference: ER/PR + Her2-)				
Triple Negative	1.48 [1.21, 1.83]	<0.001	1.44 [1.16, 1.77]	0.001
Other combinations or unknown	1.42 [1.25, 1.61]	<0.001	1.38 [1.21, 1.57]	<0.001
**Comorbidity Index** (Reference: 0)				
1	0.79 [0.63, 0.93]	0.004	0.90 [0.76, 1.06]	0.212
≥2	0.84 [0.71, 1.00]	0.051	1.04 [0.86, 1.25]	0.697
**Mammography** (Reference: No)				
Yes	0.47 [0.39, 0.57]	<0.001	0.49 [0.40, 0.60]	<0.001

### Factors associated with risk of death.

The Cox proportional hazards regressions are presented in Table 4, and were calculated controlling for age, marital status, region of residence, comorbidity index, health insurance, biomarkers, stage, and mammography screening. The proportional hazards assumption was examined [37], including all variables: age, marital status, region of residence, comorbidity index, health insurance, biomarkers, mental health, and mammography screening. The global Schoenfeld residual test was applied, indicating that the proportional hazards assumption was not met (p < 0.001). Then, we performed a Schoenfeld test for each variable and identified biomarker status as the covariate that violated the proportional hazards assumption. Therefore, the Cox model was stratified by biomarker status. The model was stratified by the biomarker variable, controlling for the other variables. Following the team stratified by biomarkers, the proportional hazards assumption was met (p = 0.338).

**Table 4:** Cox Regression Model, stratified by the variable biomarkers.

	Unadjusted		Adjusted	
	**HR [95% CI]**	**P value**	**HR [95% CI]**	**P value**
**Anxiety and Affective Disorders** (Reference: No)				
Yes	0.85 [0.64, 1.13]	0.266	1.01 [0.76, 1.35]	0.0936
**Age** (Reference: 50–59)				
60-69	1.06 [0.87, 1.30]	0.563	1.15 [0.94, 1.43]	0.180
≥70	1.85 [1.54, 2.22]	<0.001	1.94 [1.56, 2.42]	<0.001
**Marital Status** (Reference: Unmarried)				
Married	0.61 [0.52, 0.70]	<0.001	0.76 [0.65, 0.88]	<0.001
**Health Region** (Reference: Metro)				
Arecibo	0.72 [0.56, 0.93]	0.011	0.67 [0.52, 0.87]	0.002
Bayamon	0.80 [0.63, 1.01]	0.059	0.78 [0.61, 0.99]	0.044
Caguas	0.65 [0.51, 0.83]	0.001	0.63 [0.49, 0.81]	<0.001
Fajardo	1.01 [0.70, 1.46]	0.961	0.94 [0.65, 1.37]	0.761
Mayagüez	0.81 [0.65, 1.01]	0.062	0.78 [0.62, 0.98]	0.035
Ponce	0.67 [0.52, 0.85]	0.001	0.67 [0.48, 0.79]	<0.001
**Health Insurance** (Reference: Medicaid)				
Medicare	0.74 [0.61, 0.88]	0.001	0.61 [0.49, 0.76]	<0.001
Medicare-Medicaid	1.14 [0.93, 1.40]	0.214	1.00 [0.79, 1.26]	1.000
Private	0.59 [0.48, 0.74]	<0.001	0.67 [0.53, 0.83]	<0.001
**Comorbidity Index** (Reference: 0)				
1	1.11 [0.91, 1.35]	0.290	1.13 [0.93, 1.38]	0.227
≥2	1.55 [1.30, 1.86]	<0.001	1.48 [1.21, 1.80]	<0.001
**Mammography** (Reference: No)				
Yes	0.51 [0.42, 0.62]	<0.001	0.69 [0.56, 0.84]	<0.001
**Tumor Markers** (Reference: ER/PR + Her2-)				
Triple Negative	3.13 [2.54, 3.86]	<0.001	n/a	n/a
Other combinations or unknown	1.69 [1.44, 1.99]	<0.001	n/a	n/a
**Stage at Diagnosis** (Reference: Early)				
Late	3.62 [0.12, 4.21]	<0.001	3.39 [2.91, 3.95]	<0.001

After adjusting for potential confounders, having a diagnosis of anxiety or affective disorder did not increase the risk of death within 5 years (AHR = 1.01, 95% CI: 0.76, 1.35; p = 0.094). Married women had a statistically significant lower risk of death within 5 years (AHR = 0.76, 95% CI: 0.65, 0.88; p < 0.001). Lower risk of death was observed for users of private insurance and Medicare, and this finding was statistically significant (p < 0.05). On the other hand, comorbidity indexes, triple-negative biomarkers, and late stage are significantly associated with a higher risk ratio or likelihood of dying within the established period (p < 0.05).

## Discussion

The relationship between breast cancer and mental health conditions has been documented globally, including in studies conducted in the continental United States [2,6,23,26,29]. However, there is a significant gap in research addressing this intersection within women living in Puerto Rico. To our knowledge, this is the first study in Puerto Rico to use secondary data sources to examine breast cancer screening and diagnostic patterns among women with anxiety and affective disorders. By leveraging linked cancer registry and health insurance claims data, this study analysis offers new insights into how mental health conditions may impact or be impacted by access to healthcare services, as exemplified in screening behaviors and diagnostic outcomes in this understudied population.

One of the study questions was whether having a mental health diagnosis, specifically anxiety or affective disorders, impacts breast cancer screening. Results showed a prevalence of anxiety and affective disorders of 8%, comparable to that of the general population of Puerto Rico, according to Canino et al (2019) but lower than the estimates of the Behavioral Risk Factors Surveillance System [32,33]. These differences raise questions as to whether it is the result of lack of access or lack of effective identification and diagnosis of mental health conditions. Further research is needed to answer these questions.

A diagnosis of anxiety or affective disorder emerged in this study as being associated with getting mammography as recommended in the clinical guidelines [25]. One explanation may be that to get mental health diagnoses, women must access the health care system, which could mean that these medical encounters turned into opportunities for screening for breast cancer [25]. These findings align with the idea proposed by Cunningham et al. (2015) that medical encounters provide an opportunity to address risk factors and that having comorbidities (including mental health conditions) could require consistent healthcare visits, which in turn may lead to screening opportunities [3,23,38]. Others have found no association among more common disorders, such as depression or anxiety, and mammography compliance [5]. However, associations with adherence to mammography, stage at diagnosis, and cancer treatment have been identified to be related to severe mental health conditions, for example, schizoaffective disorder [5,27], which implies the need for further research about the experience of Puerto Rico. This study excluded serious mental health illnesses such as schizophrenia, alcohol dependence, or other substance dependences, among others, which may have an impact on compliance with mammography use, as encountered by other researchers [3,27,39]. Since we identify anxiety and affective disorders based on insurance claims, we only capture women with diagnosed conditions who accessed care. This likely underestimates the true prevalence of mental illness in the study population. As we did not include women with these conditions who did not seek care, we could have underestimated the prevalence of these conditions. We may also have introduced surveillance bias, as women with greater healthcare utilization may have been more likely both to receive a mental health diagnosis and to undergo breast cancer screening.

There was also interest in responding to the question of whether there was a relationship between having comorbid anxiety or affective disorders upon getting a late-stage breast cancer diagnosis. Findings in this study showed no significant relationship with stage at diagnosis. Similarly to studies in London, UK, which did not show an association between the stage at time of diagnosis and anxiety or depression [40]. Results showed no statistically significant relationship with the risk of survival, in response to the question, “Does having anxiety or affective disorders impact 5 years of survival?” These findings are important, and they can be interpreted as evidence that breast cancer screening awareness measures are reaching different segments of the population. They may also indicate that healthcare providers are effectively using each patient’s encounter to promote screening services. Mental health conditions can be developed or diagnosed at any time during the process [41], still it is important to consider a connection of treatment impacting mental health [42]. Notwithstanding, it is vital to continue researching, considering areas that have not been explored, such as the impact of having a breast cancer diagnosis and developing a mental health illness [2,43] as well as the impact of severe mental health conditions on breast cancer screening, treatment, and survival in Puerto Rican women.

Additionally, our findings demonstrated that factors such as age, marital status, health insurance, comorbidities, and biomarkers may also influence screening services such as mammography, thus impacting timely cancer diagnosis. It is essential to consider other age-related variables that may affect screening behavior and quality of life, such as mobility, cognitive decline, or healthcare access. One of the risks of developing cancer, understandably, is age, however, concerning Puerto Rico it is an essential factor to consider. The population of Puerto Rico is rapidly aging [44], representing a challenge for public health as well as for the health system, with a shortage of physicians and the economic crisis [45,46].

Marital status was also associated with screening behaviors and stage at diagnosis; being married was significantly associated with a ~ 14–15% lower likelihood of being diagnosed at a late stage. This might be due to better social support, healthier lifestyles, and increased healthcare access [18,39]. Married women have been shown to have a higher likelihood of diagnosis, which may be associated with having an additional support structure, having, in turn, a better lifestyle, diet, exercise, and access to health care. Conversely, other studies related being married to delayed screening, due to fear of the impact of a positive diagnosis on their marriage [47,48]. This suggests cultural factors that should be explored in future studies, as well as the need to consider sociological analyses of the social roles assigned to women applied to the experience of breast cancer.

Medicaid enrollees had less access to mammography, according to our study findings. This is consistent with studies in the mainland United States, where Medicaid enrollees and uninsured women face greater challenges in timely breast cancer screening and treatment [13,14]. One question emerges from these findings: whether other elements have an additional impact, such as distance to services or referral from primary care physicians to screening. Furthermore, Medicaid beneficiaries with comorbid mental health conditions are at even higher risk of inadequate screening and higher mortality rates [14].

Clinical factors such as biomarkers and comorbidities emerged, as expected, as a significant factor for assessing risk, defining prognosis, and impacting survival [49]. This calls for further investigations concerning the experiences of women in Puerto Rico. But comorbidities, particularly considering as previously stated, the challenges faced by Puerto Rico’s health system, may impact access to much-needed services.

### Limitations.

This study has several limitations. First, the retrospective cohort design relied on secondary data sources, which may introduce bias [50]. Data were derived from linking the Puerto Rico Central Cancer Registry to health insurance claims, combining validated cancer surveillance records with administrative claims primarily intended for billing [51]. While registry data are subject to rigorous quality control, claims data may include diagnostic coding errors, underreport mental health conditions, or result in misclassification. Nonetheless, the registry receives approximately 90% of claims from health insurers in Puerto Rico, strengthening the reliability of the linked dataset. Second, the analysis excludes women without health insurance coverage (ranged from 5.2% in 2012 to 4.6% in 2016) [33] and those receiving care through the Veterans Health Administration, which limits the generalizability of the findings to all women in Puerto Rico. Since our study only included insured women from the linked claims database, the results may not be generalizable to uninsured patients, who might have more difficulty accessing breast cancer screening. These groups may differ in their health behaviors, access to care, and utilization of screenings. Beyond its limitations in this study, an important system-level mechanism stands out: contact with the health system and continuity of care can influence participation in early detection programs and affect cancer treatment outcomes. Future research should expand on that possibility and explore mixed and prospective designs to validate and deepen the information on mental health conditions and breast cancer.

## Conclusion

Our study found that anxiety and affective disorders were associated with an increased likelihood of breast cancer screening among Puerto Rican women aged 50 and older. This finding may suggest that these mental health conditions may act as a secondary effect of increased medical encounters in this context. This is a noteworthy finding given the limited research examining the intersection of mental health and cancer screening behaviors in this population as a starting point on the discussion. As the burden of both breast cancer and mental health conditions grows, it is essential to consider the broader implications for healthcare delivery. Future research should explore the impact of mental health status following a breast cancer diagnosis, as well as the quality of care and life experienced by cancer patients living with co-occurring mental health disorders. Diverse methodological approaches may help ensure the inclusion of women who lack health insurance or access to primary care in future studies to improve cancer care. These insights are critical to informing culturally relevant, integrated approaches to cancer prevention and survivorship care in Puerto Rico and similar settings.
